# Wireless non-invasive continuous respiratory monitoring with FMCW radar: a clinical validation study

**DOI:** 10.1007/s10877-015-9777-5

**Published:** 2015-09-30

**Authors:** K. van Loon, M. J. M. Breteler, L. van Wolfwinkel, A. T. Rheineck Leyssius, S. Kossen, C. J. Kalkman, B. van Zaane, L. M. Peelen

**Affiliations:** 1 Department of Anesthesiology, University Medical Center Utrecht, Mailstop Q 04.2.313, P.O. Box 85500, 3508 GA Utrecht, The Netherlands; 2 Department of Intensive Care, ZGT Hospital, Almelo, The Netherlands; 3 Radar Technology at TNO, The Hague, The Netherlands; 4 Julius Center for Health Sciences and Primary Care, University Medical Center Utrecht, Utrecht, The Netherlands

**Keywords:** Radar, Respiratory rate, Monitoring, Remote

## Abstract

Altered respiratory rate is one of the first symptoms of medical conditions that require timely intervention, e.g., sepsis or opioid-induced respiratory depression. To facilitate continuous respiratory rate monitoring on general hospital wards a contactless, non-invasive, prototype monitor was developed using frequency modulated continuous wave radar. We aimed to study whether radar can reliably measure respiratory rate in postoperative patients. In a diagnostic cross-sectional study patients were monitored with the radar and the reference monitor (pneumotachograph during mechanical ventilation and capnography during spontaneous breathing). Eight patients were included; yielding 796 min of observation time during mechanical ventilation and 521 min during spontaneous breathing. After elimination of movement artifacts the bias and 95 % limits of agreement for mechanical ventilation and spontaneous breathing were −0.12 (−1.76 to 1.51) and −0.59 (−5.82 to 4.63) breaths per minute respectively. The radar was able to accurately measure respiratory rate in mechanically ventilated patients, but the accuracy decreased during spontaneous breathing.

**Electronic supplementary material:**

The online version of this article (doi:10.1007/s10877-015-9777-5) contains supplementary material, which is available to authorized users.

## Introduction

Several observational studies support the idea that in-hospital deaths can be prevented by early recognition of abnormal vital signs as a marker of physiological decline [1–3]. However, timely recognition of abnormal vital signs on general hospital wards remains a major challenge as nurses have only limited time to observe their patients and perform systematic evaluations. Changes in respiratory rate (RR) are an important indicator of physiological decline and were found to be present as early as 6–24 h prior to adverse events such as cardiac arrest [2–6]. Also, altered RR is one of the first symptoms of numerous diseases that could benefit from timely intervention, hereby preventing further deterioration. Examples are increased RR in pneumonia, sepsis and cardiogenic shock; and decreased RR with opioid overdose. Nevertheless, clinicians still underestimate the importance of a change in RR and it is probably the least observed and recorded vital sign on general hospital wards [7]. Currently, the usefulness of RR is limited due to its large inter-observer variability, as RR is counted by observing chest wall movements [8, 9]. More importantly, observers (i.e. nurses) can only provide intermittent assessments, as RR is typically estimated only two or three times a day. This leaves a patient’s respiration unmonitored for long periods of time (typically 8 h or more) and any physiological decline that occurs between these RR observations can easily go unnoticed.

Recently, the Opal radar system was developed to detect vital signs (RR and heart rate) in humans (TNO laboratories, The Hague, Netherlands). The radar uses frequency modulated continuous wave (FMCW) radar to obtain a periodic signal by frequency modulating a continuous signal that is mixed with an echo. The radar is therefore able to obtain information on movement in multiple directions (e.g. the expansion of the chest during breathing), based on the principle of integrating a transmitted and received signal [10]. The radar system was previously found to be capable of detecting breathing patterns non-invasively in human volunteers [10]. However, rigorous scientific evaluation has to be conducted before introducing a new technical device into clinical practice. The primary aim of this study was therefore to determine whether this prototype radar system is able to reliably measure RR in patients during mechanical ventilation and spontaneous breathing.

## Materials and methods

### Study population

We aimed to study the FMCW radar in postoperative surgical patients during mechanical ventilation (MV), where RRs and tidal volumes are exactly known, and during spontaneous breathing. We included adult patients at the University Medical Center Utrecht, the Netherlands, who underwent elective robotic assisted laparoscopic prostatectomy or hysterectomy and received post-operative ventilation on the postoperative anesthesia care unit (PACU) (as per clinical protocol). Patients with an implantable cardiac device, or on renal replacement therapy, or who refused participation or who were unable to give informed consent, were excluded. The institutional review board (IRB) of the University Medical Center Utrecht reviewed the study protocol and found that it was not subject to the Dutch act on “medical research involving human subjects”. Therefore, the IRB waived the need for informed consent, but we decided to request written informed consent from all participants in order to promote transparent information provision to patients. The study was conducted in accordance with the moral, ethical, and scientific principles governing clinical research as set out in the Declaration of Helsinki [11] and good clinical practice.

### Study design and measurements

**Fig. 1:**
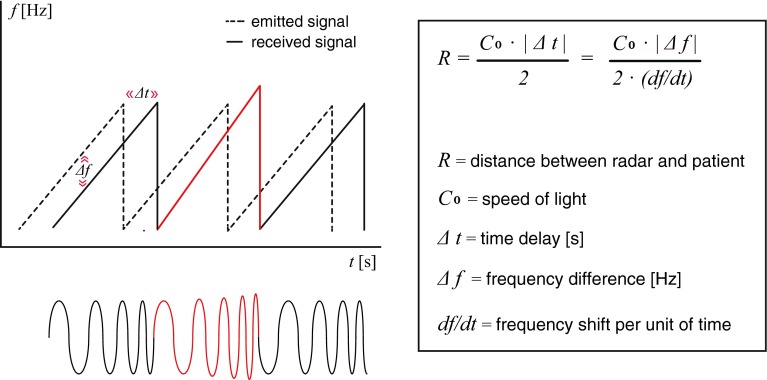
The principle of frequency modulated continuous wave (FMCW) radar. The frequency shift (∆*f*) of the emitted radar waves over time enables us to determine the distance to the patient. Patient breathing (or movement) changes the amplitude (energy) of the reflected signal (the *red line*) and the distance (*R*)

This study was a diagnostic cross-sectional observational study in which patients were monitored after surgery during recovery in the PACU to conform with current guidelines for postoperative care. The non-invasive FMCW radar signals (with the device mounted to the ceiling above the patient, see Fig. 1) were recorded simultaneously with those from the reference standard. During MV the reference standard was the pneumotachograph from the ventilator (Raphael, Hamilton Medical AG 2005, Bonaduz, Switzerland). The pneumotachograph has an accuracy of ±1 breath/min. During spontaneous breathing the reference standard was a capnograph (Compact S5, Datex Ohmeda, Inc., Helsinki, Finland) with end-tidal carbon dioxide (etCO2) sampling lines (Smart CapnoLine Plus, Oridion Medical 1987 Ltd, Jerusalem, Israel). The capnograph has an accuracy of ±1 breath/min in the range 4–20 breaths/min. In addition, patients were observed during study measurements by one of the authors (KvL), who recorded time-stamped patient movements and staff activity around the bed. Nursing and medical staff was blinded to the study measurements with the FMCW radar.

### FMCW radar principle

The FMCW radar system is a small box sized 7.5 by 10 cm mounted to the ceiling above the bed. This prototype radar operates on a frequency of 9–10 GHz with an effective isotopically radiated power of 14 mW, which is well within restrictions for human exposure to electromagnetic fields [12]. The emitted electromagnetic radio waves (frequency of 9.5 GHz) propagate to the patient, are reflected, and arrive back at the radar with a time difference (∆*t*). This causes a frequency difference between emitted and received signal due to the fact that the radar changes the frequency of the radio waves over time, consistent with a sawtooth pattern in Fig. 1. This frequency shift (∆*f*) over time enables us to measure the time difference between emitted and received signal (∆*t*), and obtain the distance (*R*) between the radar and the patient. The relation between distance and time difference is given in the formula in Fig. 1. Patient breathing (or moving) changes the amount of reflected energy to the radar (resulting in amplitude changes in the radar output signal) and the distance to the patient (resulting in phase changes in the radar output signal). Additional open source technical information about FMCW radar is available online [13] and in Supplement 1.

### Signal analysis

The raw data recorded by the FMCW radar system consisted of numerical indicators for the amplitude and phase from a certain spatial range *R*. The area under the radar was divided into horizontal planes (range bins) of 21 cm height. Patient movement (e.g. chest wall movement for respiration) typically extended over 2 or 3 range bins. By selecting the range bin with the highest power in the frequency band up to 40 Hz, artifacts were largely neglected and the optimal range bin was selected. As the patient could change position several times, the ‘optimal’ range bin was selected every minute. The amplitude and phase of the echoed radar waves contained information about movement of the patient inside the selected range bin. In this study we only used the amplitude information.

RRs were determined with Fast Fourier Transform (FFT) [14]. As we aimed to conduct our analysis both on raw data (complete dataset) and after removal of voluntary movement artifacts (artifact reduced dataset), a dedicated algorithm was written for artifact detection. We first visually identified differences between consecutive points during rest and during other activities, using the observations on patient and staff movement. Subsequently, a fixed threshold (electric potential of 0.005 V) was applied for differences between consecutive amplitude points. The number of artifacts was defined as the number of points that exceeded this threshold. During MV FFT was applied on each 60-s time sample, as the reference standard was also retrieved from a 60-s time sample, to obtain the corresponding RR by taking the maximum peak in the frequency window. During spontaneous breathing FFT was applied over a 30 s time interval, as it was expected that during spontaneous breathing the number of artifacts would increase due to activities of nursing staff or obvious patient movement. Out of two retrieved RRs, the one with the lowest number of artifacts was used for further analysis. In case both RRs had a similar amount of artifacts, the RR used in further analysis was calculated as the average of these RRs. For the reference standards we did not develop a specific algorithm for artifact detection, but used the RRs as provided by the pneumotachograph and the capnograph respectively.

### Outcomes

The primary outcome was the RR. As bradypnoea is a serious life threatening condition with a narrow diagnostic window, we considered a RR with limits of agreement (LoA) of −2 to 2 breaths per minute acceptable. The secondary outcome was accuracy in detecting abnormal respiration (RR < 12 or >20 breaths per minute) according to the early warning score definitions [15].

## Statistics

The paired time series of RR measurements (one data point every minute) derived from FMCW radar and the reference standard were compared using the Bland and Altman method for repeated measurements [16]. In this method the variance for differences between the average difference across patients is corrected for the number of measurements per patient. We determined the bias (mean difference) and 95 % LoA’s (±1.96*SD) for the complete and reduced (after removal of artifacts) dataset. For this type of studies no formal rules for sample size calculations are available. We considered 250 measurement pairs for respiratory rate to be sufficient for adequate estimation of the repeated measures Bland–Altman bounds. Abnormal breathing rates were defined as a respiratory rate below 12 breaths and above 20 breaths per minute. Sensitivity, specificity, positive predictive value, and negative predictive value for abnormal breathing were calculated. Corresponding confidence intervals were produced with the Wilson Score method without continuity correction. Additionally, a Clarke error grid analysis (EGA) was performed to quantify clinical accuracy and the consequences for clinical decision making [17]. The analyses were conducted using MatLab (The MathWorks^®^, Inc) and the public domain statistical software ‘R’ version 2.15.3 (URL: http://www.R-project.org).

## Results

From May 2011 to August 2011, 796 min were recorded during MV and 521 min during spontaneous breathing in eight patients who gave informed consent for study participation. For MV, 796 complete RR measurement pairs were available, and 351 (67 %) for spontaneous breathing. Missing data of spontaneous breathing measurements consisted of 169 min with missing capnography measurements and one missing FMCW radar measurement. The number of observations on each patient ranged from 17 to 170 during MV and 23–70 during spontaneous breathing. Characteristics for the individual patients are shown in Table 1. None of the patients had pulmonary comorbidities or abnormalities of the chest wall. All patients received analgesia with acetaminophen and intravenous morphine for postoperative pain during their stay on the PACU. During spontaneous breathing patients received supplemental oxygen (1–3 L/min). In 98.3 % of all SpO2 readings the SpO2 was ≥95 %, and none of the patients had SpO2 readings below 90 %.

**Table 1:** Characteristics of individual patients and measurements during monitoring with frequency modulated continuous wave radar in postoperative patients

					Mechanical ventilation		Spontaneous breathing		
Patient	Gender (m/f)	Age (years)	BMI (kg m^2^)	Surgical procedure	Tidal volumes during MV mean (SD)^a^	Radar measures (min)	Reference measures (min)	Radar measures (min)	Reference measures (min)
1	M	53	26.0	Prostatectomy	768 (48)	112	112	31	30
2	M	54	31.2	Prostatectomy	903 (199)	141	141	26	23
3	M	62	24.5	Prostatectomy	697 (20)	58	58	60	56
4	M	58	24.5	Prostatectomy	695 (67)	170	170	42	37
5	F	34	31.7	Hysterectomy	586 (86)	156	156	77	67
6	F	36	19.4	Hysterectomy	651 (82)	72	72	102	34
7	M	63	25.3	Prostatectomy	678 (110)	17	17	143	70
8	F	58	22.7	Hysterectomy	413 (49)	70	70	39	34

*M* male, *F* female, Surgical procedures were robot assisted laparoscopic procedures

^a^ *MV* mechanical ventilation

### Respiratory rate during mechanical ventilation

**Fig. 2:**
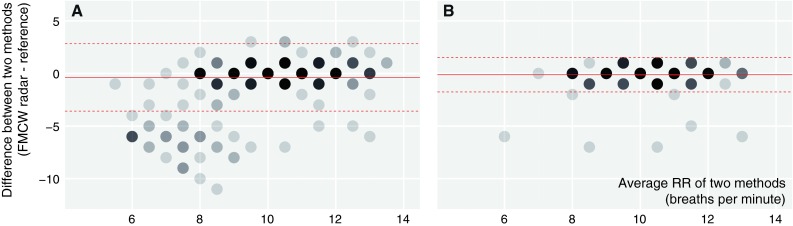
Bland and Altman plots for complete (**a**) and movement artifact reduced (**b**) datasets for respiratory rate during mechanical ventilation with 1 (*filled circle*) to 10 (*filled circle*) measurement pairs

Bias and 95 % LoA from comparisons between the radar and the pneumotachograph are corrected for the number of observations on each patient and shown in Table 2. After artifact removal during MV, 441 min could be analyzed. The bias (RR radar minus RR pneumotachograph) was −0.12 breaths per minute with a 95 % LoA of −1.75 to 1.51 breaths per minute. Bland and Altman plots for the complete and artifact reduced datasets are shown in respectively Fig. 2a and b.

**Table 2:** Primary outcome: respiratory rates during frequency modulated continuous wave radar monitoring in postoperative patients

Study phase and analysis	Number of measurement pairs	Bias	SD	Lower 95 % LoA^b^	Upper 95 % LoA^b^
*Mechanical ventilation*					
Complete dataset	796	−0.37	1.64	−3.58	2.83
Reduced dataset^a^	441	−0.12	0.83	−1.76	1.51
*Spontaneous breathing*					
Complete dataset	351	−1.21	3.57	−8.20	5.78
Reduced dataset^a^	185	−0.59	2.67	−5.82	4.63

^a^Dataset after elimination of movement artifacts

^b^ *LoA* limits of agreement

### Respiratory rate during spontaneous breathing

**Fig. 3:**
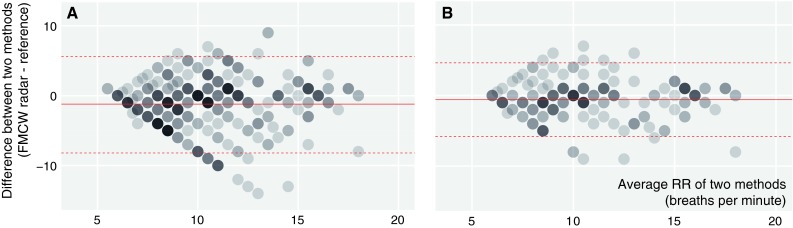
Bland and Altman plots for complete (**a**) and movement artifact reduced (**b**) datasets with respiratory rate during spontaneous breathing with 1 (*filled circle*) to 10 (*filled circle*) measurement pairs

During spontaneous breathing, after artifact removal, the RR measured by the radar was on average −0.59 breaths per minute with a 95 % LoA of −5.82 to 4.63 breaths per minute (Table 2). Bland and Altman plots for the complete and reduced dataset displayed in Fig. 3 indicate that the difference between FMCW radar and capnography depends on the average breathing rate. For respiratory rates below 10 and above 15 breaths per minute differences between FMCW radar and capnography are within the 95 % LoA, whereas between 10 and 15 breaths per minute the monitors differ considerably

### Diagnostic accuracy for abnormal breathing

**Fig. 4:**
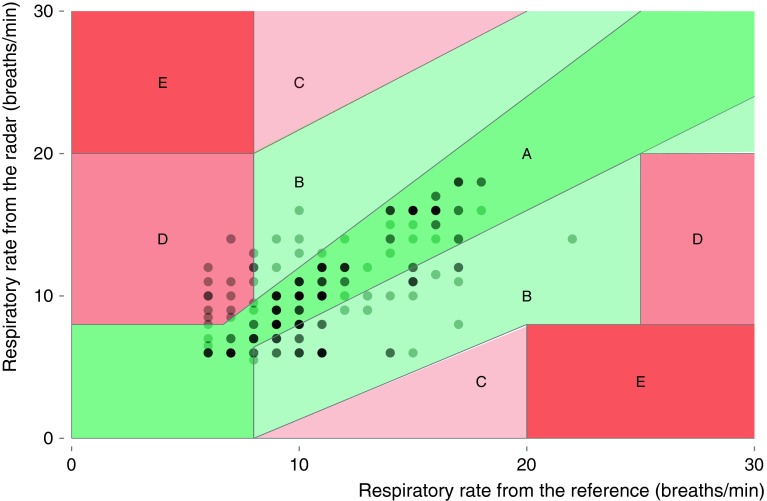
Clarke error grid to quantify clinical accuracy of the respiratory rate measurements by radar. Region (*E*) represent those points where tachypnoea and bradypnoea are confused, region (*D*) indicates a potentially dangerous failure to detect bradypnoea or tachypnoea, in region (*C*) are points leading to unnecessary treatment, region (*B*) contains points outside 20 % of the reference but not leading to unnecessary treatment, region (*A*) are points within 20 % of the reference measurement

Tachypnoea was defined as a RR above 20 breaths per minute. During spontaneous breathing only 2 min with tachypnoea were recorded by the capnograph. Therefore we were unable to calculate diagnostic accuracy for tachypnoea. Bradypnoea occurred frequently during spontaneous breathing, a RR below 12 breaths per minute was present in 64 % of all RR minutes in the complete dataset analysis. The sensitivity, specificity, PPV and NPV are shown in Table 3. After removal of artifacts, the FMCW radar had a positive predictive value (PPV) of 86 % (79–92 %), and a negative predictive value (NPV) of 75 % (64–85 %) for detection of bradypnoea. Ninety-one percent of respiratory rate measurements in the error grid analysis are within region A and B (Fig. 4), respectively within 20 % of the reference measurement, or outside 20 % of the reference but not leading to unnecessary treatment. Region D, indicating a potentially dangerous failure to detect bradypnoea contains nine percent of the measurements. None of the measurements were in region C or E, which means that none of the measurements would lead to unnecessary treatment or confusion between tachypnoea and bradypnoea.

**Table 3:** Secondary outcome: diagnostic accuracy for bradypnoea (respiratory rate <12 breaths per minute) during spontaneous breathing

Study analysis	True positives	False positives	True negatives	False negatives	Sensitivity (95 % CI)	Specificity (95 % CI)	PPV^c^ (95 % CI)	NPV^d^ (95 % CI)
SB^a^ (complete dataset)	180	56	69	45	80 (74–85)	55 (46–64)	76 (71–81)	61 (51–69)
SB^a^ (reduced dataset^b^)	104	17	48	16	87 (80–92)	74 (63–84)	86 (79–92)	75 (64–85)

Values are number and proportion

^a^ *SB* spontaneous breathing

^b^Dataset after elimination of movement with artifacts due to obvious movement

^c^ *PPV* positive predictive value

^d^ *NPV* negative predictive value

## Discussion

We studied the ability of a prototype FMCW radar system to measure RR in patients during MV and during spontaneous breathing. After elimination of obvious movement artifacts, the FMCW radar accurately measured RR in patients during MV with a deviation within two breaths of the reference standard. During spontaneous breathing, while the patient is awake and physically active, the accuracy of the FMCW radar to detect RR every minute was outside our acceptable limits. Presumably, the clinical study setting, the pharmacological (e.g. opioid administration) and the surgical treatment, all contributed to the differences between the FMCW radar- and capnography RR measurements during the spontaneous breathing phase. Interestingly, the deviation of the FMCW radar RR measurements strongly depended on the average RR. The radar appeared to be more accurate during episodes with low RR. One possible explanation is that patients in deep sleep, shortly after anesthesia and after opioid administration, lie still and breathe slowly. In general, patients with an opioid breathing pattern show bradypnoea with large tidal volumes and larger chest wall movement [18]. This probably explains the high accuracy of FMCW radar during episodes with low breathing rates, with a sensitivity of 87 % as well as a positive predictive value of 86 % for detection of bradypnoea. This finding is very important, since during bradypnoea even small deviations in RR measurements are clinically relevant.

The accuracy of the FMCW radar improved when we excluded 1-min epochs with obvious movement artifacts. These artifacts were characterized by a large fluctuation of the reflected energy (amplitude) within a least five range bins (Supplement 2). Thus, the FMCW radar is not able to detect RR accurately when there is obvious patient (or provider) movement in the same range bin as the chest wall movements. When considering how these artifacts may influence the feasibility of FMCW radar to monitor RR in patients on a general hospital ward, there are several scenarios to consider. When a caregiver or family member in close vicinity of the patient is responsible for the movement artifacts, this person will probably notice apnea/unresponsiveness, if present. Also, when radar artifacts are caused by active patient movements, these artifacts imply that the patient is alive and breathing. It should be noted however that there are, two possibly life-threatening exceptions to this latter situation: extreme agitation due to hypoxemia and grand mal seizure activity. Furthermore, with any technique that relies on measuring chest wall movements, detecting such movement does not guarantee displacement of air. Therefore the FMCW radar might have difficulty recognizing progressive upper airway obstruction. In this specific situation chest wall movements will be intact without air movement. This problem can potentially be solved if future radar algorithms are optimized to detect paradoxical movement of chest wall and abdomen, and other opioid- induced changes of the breathing pattern (e.g. increased intercostal contribution, increased tidal volume, and increased respiratory variability) [18, 19] by pattern recognition techniques [20].

As far as we know, this is the first study with FMCW radar to measure RR in postoperative surgical patients. A variety of medical radars to measure RR and heart rate have been described. Most of these studies used continuous wave (CW) Doppler radar or impulse ultra wide band (UWB) radars. See for a detailed overview the FFI report Medical Radar literature overview by Aardal [21]. Although CW radars are sensitive in detecting time varying physiological phenomena, they do not provide range information which FMCW and UWB radars do provide. While some of these radars are studied in hospital environments, most experiments were conducted under controlled laboratory conditions. Vasu [22] studied RR overnight with a non-contact Doppler radar in ten patients without sleep disorders and compared these measurements to expert annotations. Other (UWB) radars that are being tested in hospital environments showed correct heart rate and RR measurements [23]. In our study only eight patients were included, but we provide a large number of data points during both MV (796 data points) and spontaneous breathing (351 data points) with both the FMCW radar and the reference device.

## Limitations

When interpreting the findings of this study some limitations should be taken into account. First, since a predefined algorithm for analyzing FMCW radar data was not available, we may have selected a suboptimal signal-processing algorithm. In this study we used FFT to translate the amplitude information of the radar waves into RRs. FFT uses the symmetry and periodicity of the signal to create a frequency window. Unlike during MV, the breathing pattern during spontaneous breathing in awakening patients is variable, even more evident than in awake patients, with alternately deep and shallow breaths, and fast and slow breathing (dependent on alertness, speech and emotional state). Probably, this is also the case for the observed RR differences in the range 10–15 breaths per minute. In that situation, the peak energy in the frequency domain will disperse into a wider frequency band, making ‘exact’ RR estimation difficult and potentially unreliable. Alternative signal processing techniques as wavelet transform (WT) could be used in future evaluations [24]. We cannot exclude that this may improve the performance of FMCW radar. Another possible limitation of the current algorithm is the selection of the ‘optimal’ range bin depending on the highest power per range bin. Range bins with high frequencies (i.e. due to artifacts) were excluded before selection of the optimal range bin. It is conceivable that this selection order may have induced suboptimal range bin selection in some situations. Furthermore, for certain range bins the amplitude of the breathing signal shows two maxima per time period, that could indicate that different body parts move inside the same range bin, or that the patient oscillates through the center of the range bin, impeding the extraction of the correct peak in the frequency window. Another restriction of the signal analysis is that the literature is silent on thresholds for the detection of artifacts. We chose a fixed artifact threshold at 0.005 V based on the amplitude waveforms during observed movements. A lower threshold (<0.005 V) could eliminate more artifacts at the expense of limiting the number of available valid RR measurements. On the other hand, improved artifact elimination analysis could result in a higher proportion of epochs with reliable RR estimations and lower rate of false positive alarms. This is especially important in low care settings such as general hospital wards with lower nurse-to-patient ratio than recovery rooms or intensive care units.

A second limitation of our study was that our approach of rejecting 1-min epochs with excessive artifacts decreased the availability of continuous RR monitoring (Supplement 3). This procedure also made the relation between consecutive RR measurements more variable, which potentially hinders interpretation of the results from the Bland and Altman plots. For respiratory monitoring in clinical practice, slightly variable RR measurement updates are probably not detrimental, because the goal for continuous monitoring is to recognize physiological decline, which typically develops in the hours preceding a serious adverse event.

Third, it is important to consider that FMCW radar information consists of a complex time signal with two dimensions describing the echoed radar waves, namely the amplitude and phase. In this study we used only the amplitude information to estimate RRs. Incorporating the phase information as a second dimension could give additional information and can potentially increase the accuracy of the RR estimation in future studies.

### Other potential applications

**Fig. 5:**
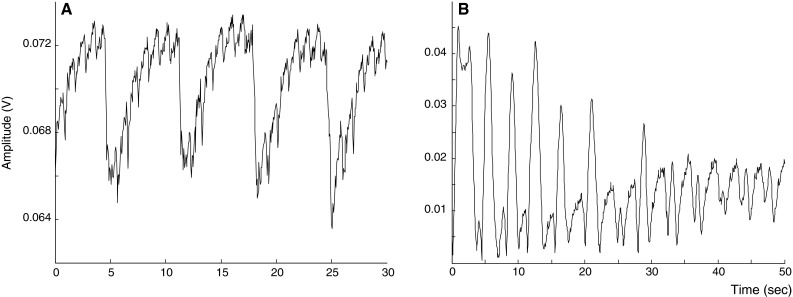
Potential features of the frequency modulated continuous wave radar. **a** Example (patient number 8) of five breaths with 31 superimposed heartbeats on the 30 s radar signal during mechanical ventilation, that correspond to the heart rate measured by pulse oximetry. **b** Example (patient number 1) of a radar trace during 50 s of spontaneous breathing with different breathing patterns potentially corresponding to the tidal volumes. Every inspiratory cycle is characterized by a first peak (chest wall expansion during inhalation), followed by a more smoothed peak (during exhalation). We presume that larger tidal volumes, thus increased chest wall expansion result in higher amplitudes of the echoed radar waves

Although our study focused on detection of RR, we noticed that the heart rate was present in some parts of the amplitude information. Figure 5a shows an example of five breaths with 31 superimposed beats on the 30 s radar signal. This corresponds with the patients’ actual heart rate of 63 beats per minute during this specific period. This is in line with findings of Aardal [25] who demonstrated the ability of FMCW radar to detect heart rate non-invasively. Future studies should determine if heart rate can be reliably determined from the raw radar data using optimized signal processing algorithms.

Another interesting issue is whether the raw amplitude radar data can also be used to estimate respiratory depth. Being able to distinguish between progression to rapid shallow breathing versus rapid deep breathing would be clinically important to assess a patient’s reserve capacity. Some patients had radar amplitude patterns that suggested this might be the case (Fig. 5b). Estimating actual tidal volumes is likely to be much more difficult, since a patient in the supine position will produce different radar amplitudes compared to the lateral position. This problem may be solved by using two different radars placed on the ceiling above the patient instead of just one. Both phenomena are subject to future study.

## Conclusion

Considering the ability of the FMCW radar system to accurately track RR during MV accurately and the possibilities for further optimizing of the signal processing algorithms, we suggest that future iterations of this wireless technique have the potential to support early recognition of physiological decline in spontaneously breathing patients on general hospital wards. However, the accuracy of RR determination during spontaneous breathing needs further refinement. Nonetheless, even with the current relatively crude movement artifact removal algorithm, enough epochs were left to track the RR trend of spontaneously breathing postoperative patients recovering from general anesthesia on a minute to minute basis. To confirm the feasibility of this radar-based technique for continuous and wireless respiratory monitoring in patients on a general hospital ward, a larger prospective cohort study is needed.

## Electronic supplementary material

Below is the link to the electronic supplementary material.

Supplementary material 1 (PDF 760 kb)

Supplementary material 2 (PDF 1136 kb)

Supplementary material 3 (PDF 711 kb)
