# Supplementary material for: Wireless non-invasive continuous respiratory monitoring with FMCW radar: a clinical validation study
Source: J Clin Monit Comput. 2015 Sep 30;30(6):797–805. doi: 10.1007/s10877-015-9777-5 (PMC5082588; doi:10.1007/s10877-015-9777-5)
Supplement: Supplementary file 1 — Supplementary material 1 (PDF 760 kb) [file 10877_2015_9777_MOESM1_ESM.pdf]

Supplementary material 1

Frequency Modulated Continuous Wave (FMCW) radar components scheme

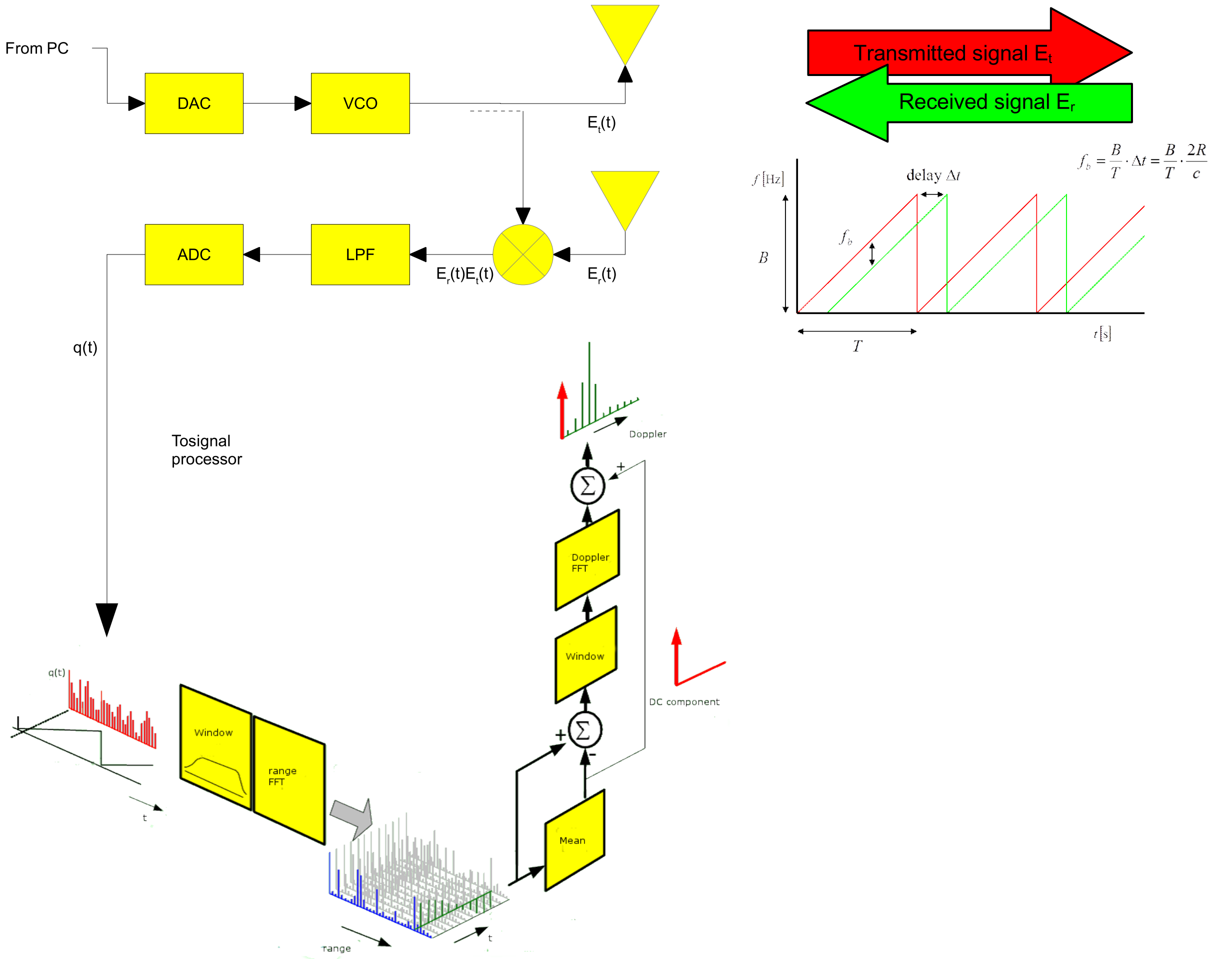

Journal: Journal of Clinical Monitoring and Computing
Authors: van Loon K, Breteler M.J.M., van Wolfwinkel L, Rheineck Leyssius A.T., Kossen S, Kalkman C.J., van Zaane B, Peelen L.M.
Affiliation: University Medical Center Utrecht
Email: K.vanLoon-3@umcutrecht.nl
