# Supplementary material for: Wireless non-invasive continuous respiratory monitoring with FMCW radar: a clinical validation study
Source: J Clin Monit Comput. 2015 Sep 30;30(6):797–805. doi: 10.1007/s10877-015-9777-5 (PMC5082588; doi:10.1007/s10877-015-9777-5)
Supplement: Supplementary file 3 — Supplementary material 3 (PDF 711 kb) [file 10877_2015_9777_MOESM3_ESM.pdf]

Supplementary material 3.
Distribution of data intervals for all patients within four datasets
(MV = mechanical ventilation, SB = spontaneous breathing)

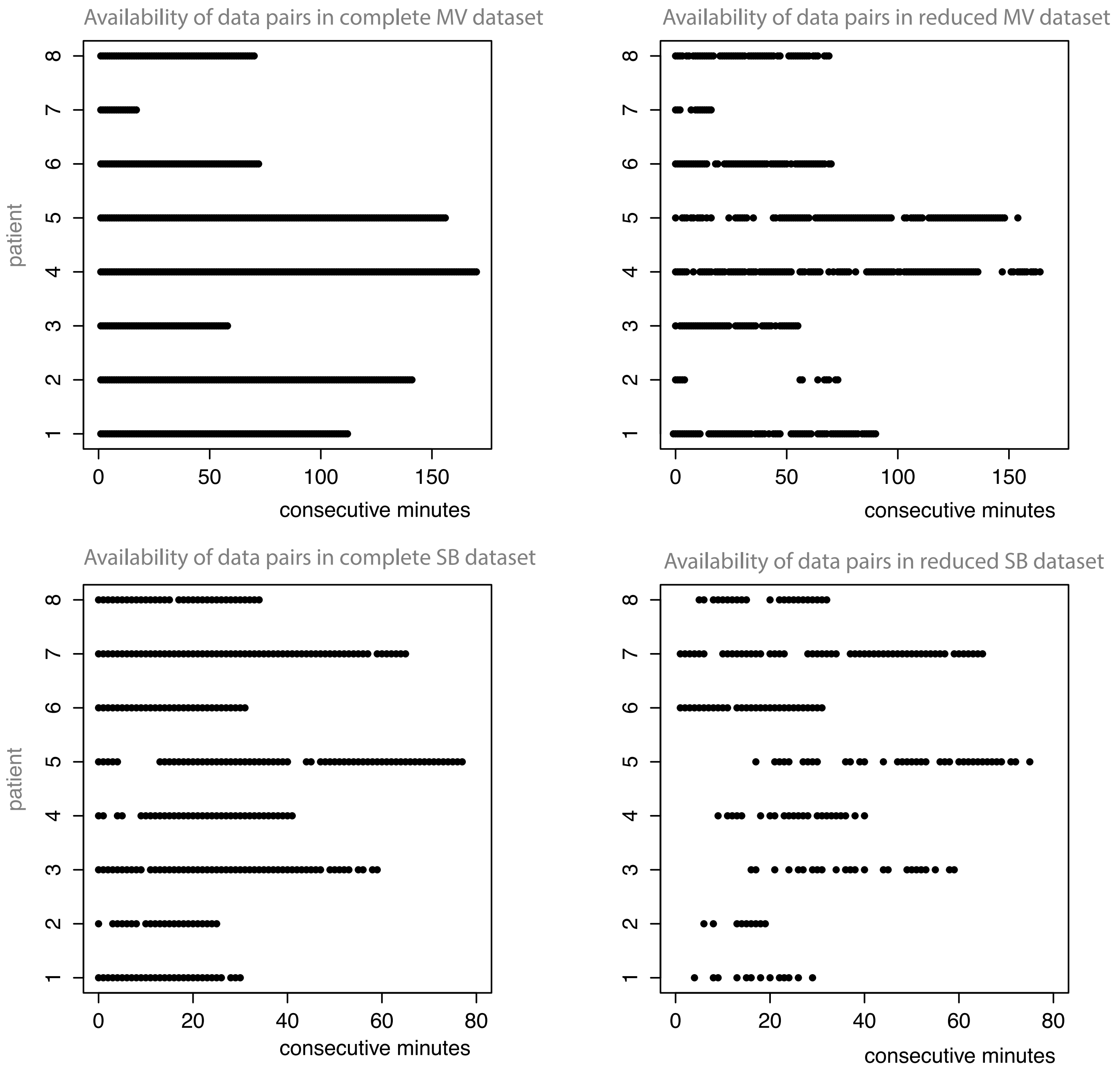

Journal: Journal of clinical monitoring and computing
Authors: van Loon K, Breteler M.J.M, van Wolfwinkel L, Rheineck Leyssius A.T, Kossen S, Kalkman C.J, van Zaane B, Peelen L.M.
Affiliation: University Medical Center Utrecht
Email: K.vanLoon-3@umcutrecht.nl
